# What's in a name? Commentary: A crisis in comparative psychology: where have all the undergraduates gone

**DOI:** 10.3389/fpsyg.2015.01856

**Published:** 2015-12-15

**Authors:** Mathias Osvath, Tomas Persson

**Affiliations:** Cognitive Zoology Group, Department of Cognitive Science, Lund UniversityLund, Sweden

**Keywords:** comparative psychology, comparative cognition, recruitment, undergraduates, graduate programs, education

Abramson recently expressed concern over the fact that the comparative psychology discipline might be in danger, as the number of academic courses bearing its name is low (Abramson, 2015). In contrast to Abramson we do not think that a fading name equals a dying science. On the contrary, it is likely a sign of a developing science and a more multifaceted education.

That a discipline's name does not contain “comparative” does not mean that comparisons are not made, or are crucial. If subscribing to the definition given by Abramson: “similarities and differences in the behavior of organisms,” it is in fact difficult to imagine an animal science without any comparisons. For example, ethology is a branch of science with inherently comparative studies of animal behavior (Burkhardt, 2005). There is no obvious monopoly on comparative methods in comparative psychology. The approach of comparing organisms is a biological one, stemming from pre-Darwinian times in such fields as comparative anatomy.

Comparisons with humans, especially, are ubiquitous in many disciplines. That such disciplines would not offer training in making valid comparisons—whatever that is—appears to be an unfounded assumption. We hope that animal sciences, along with most other sciences, also develop the students' “critical thinking skills, personal exploration, cultivating a comprehensive view of the world around them, and their ability to apply their skills in both academic and applied fields” just as well as comparative psychology does. Students can certainly learn about “analogies, homologies, subject variables, environmental variables, observation skills, etc.” in a course on e.g., animal cognition, or behavioral ecology.

The crux of the matter seems to be that Abramson lacks a place for the comparative study of behavior without reference to cognition. Still, he wants to call this psychology, and demand a place for this in introductory texts and courses on psychology. It is true that “cognition” or “cognitive” are moot and debatable terms, but so is “behavior” (Levitis et al., 2009). Cognition is a contemporary term that for us encompasses a host of phenomena concerning behaviors in organisms with a nervous system. Contrary to the author we believe that cognition is a broad concept, and involves fundamental processes.

Abramson posits that comparative cognition is based on a very specific theoretical position, including the belief that internal cognitive processes can be studied scientifically. First of all, cognitive science(s) is far from a specific theoretical position, there are numerous contradicting hypotheses. Secondly, the belief that cognition can be measured through behavior is as much a belief as the notion that evolution can by measured through observations, or that learning might be studied without assuming memory, and retrieval mechanisms. Thirdly, viewing cognition as purely “internal” is an antiquated position; contemporary cognitive theories often view cognition as a causal process where the body's interaction with the environment is essential, or is *part* of the cognitive process (Gärdenfors, 2008).

Abramson seems to think that such things as physiology and evolution are beyond the scope of comparative cognition—we cannot see this. He also appears to mean that behaviorist questions cannot be studied within a cognitive framework. If this refers to learning theories, then such an assumption is false. Learning theories are in our experience given plenty of room in courses on e.g., animal cognition.

We would share Abramsons' concerns of a disappearing body of knowledge if we thought it was true. Instead, we think that the successful methods and theories of such various fields as e.g., comparative psychology, ethology, neurocognition, developmental psychology, philosophy, and theoretical biology, are today incorporated in the education in animal cognition, as well as in other courses with different names. The study of animals' behaviors and minds has always been an eclectic enterprise.

Fractionalisation is a natural result of both the dynamics of science and the branding necessary to explain a field's content. We regret to learn that Abramson sees a need for faculties to point out the value of comparative psychology. But is this best done by distancing the subject from sister subjects? This is arguably not a fruitful way to establish the image of an important discipline. Integration is the proof that something is of use for something else.

Comparative psychology has been a discipline mainly of North American concern, and Abramson's paper is heavy with references to American conditions. In Europe there has traditionally been a focus on the biological disciplines, such as ethology (Burkhardt, 2005). One should not be surprised in not finding many programs and courses named “comparative psychology” when browsing European universities. In Sweden we call the subject “cognitive zoology,” and for pedagogical reasons we call our courses “animal cognition.” In Austria it is called “cognitive biology.” In UK one would find various names and so on. However, the curriculums of the different programs and courses overlap. Perhaps this is the case in USA too? It could of course be that the implicit definition of “comparative cognition” differs between USA and Europe, which might give Abramson right with respect to “comparative cognition” being a heavily reduced form of “comparative psychology.” In that case the cognitively inclined researchers in Europe should be worried too, as Abramson's observations would then indeed indicate a narrowing of the theoretical scope in USA. In order to judge whether this is the case, a more qualitative analysis is required.

## Funding

This work was supported by The Swedish Research Council.

### Conflict of interest statement

The authors declare that the research was conducted in the absence of any commercial or financial relationships that could be construed as a potential conflict of interest.
